# Selenium deficiency impairs host innate immune response and induces susceptibility to *Listeria monocytogenes *infection

**DOI:** 10.1186/1471-2172-10-55

**Published:** 2009-10-24

**Authors:** Chengmin Wang, Haijing Wang, Jing Luo, Yi Hu, Lei Wei, Mingxing Duan, Hongxuan He

**Affiliations:** 1 National Research Center for Wildlife Born Diseases, Key Laboratory of Animal Ecology and Conservation Biology, Institute of Zoology, Chinese Academy of Sciences, Beijing 100101, PR China; 2 The Graduate University of Chinese Academy of Science, Beijing 100049, PR China; 3 State Key Laboratory of Biomembrane and Membrane Biotechnology, Department of Biological Sciences and Biotechnology, Tsinghua University, Beijing 100084, PR China

## Abstract

**Background:**

Susceptibility or resistance to infection with *Listeria monocytogenes *correlates with Selenium (Se) deficiency in response to infection.

**Results:**

Se-deficient mouse models of listeriosis were used to study the innate immune response during the course of *L. monocytogenes *infection. Blood samples from mouse models were used for Se status. The concentration of MDA, SOD, GPx and CAT in blood has revealed that lower Se level exist in Se-deficient mice. Intestine, mesenteric lymph node, liver, spleen and brain from each mouse were to study the bacterial burden in organs. The analysis of cell types of spleen from Se-deficient mice revealed that the ability of the host to elicit a rapid recruitment and activation of systemic innate immune response to infection was to a certain extent compromised under conditions of Se deficiency. The cytokine levels in the serum and cytokine expression levels in the livers from Se-deficient mice revealed that the innate immune response of Se-deficient mice was impaired throughout the course of infection. These results suggest that innate immune response is altered by Se deficiency after infection with *L. monocytogenes*.

**Conclusion:**

In conclusion, induced susceptibility of host resistance is associated with an impaired innate immune response following infection with *L. monocytogenes *in C57BL/6 Se-deficient mice.

## Background

*Listeria monocytogenes (L. monocytogenes) *is a gram-positive bacterial rod which may cause severe infections, particularly in immunocompromised patients as well as in fetuses, neonates, and the elderly [1]. Congenital infection is the most severe form, usually presenting as granulomatosis infantiseptica, a generalized disease involving the central nervous system (CNS), the gastrointestinal tract, the respiratory system, the lymphatic system, and the kidney. Mortality from neonatal listeriosis is high and virtually 100% if left untreated [2]. *L. monocytogenes *was recognized as a foodborne pathogen since the 1980s when several outbreaks of listeriosis were identified in Europe and North America [1].

Selenium (Se) is of fundamental importance to human health. It is an essential component of several major metabolic pathways, including thyroid hormone metabolism and antioxidant enzyme defense systems. Se is incorporated as selenocysteine at the active site of a wide range of selenoproteins [3]. Under physiological conditions, the Se in selenocysteine is an extremely efficient biological catalyst. Among the selenoproteins with identified biological functions are the antioxidant enzymes glutathione peroxidase (GPX) and thioredoxin reductase (TrxR). Dietary Se is essential for a healthy immune system [4] and Se influences both the innate and the adaptive immune responses [5,6]. The effects of Se deficiency include reduced T-cell numbers and impaired lymphocyte proliferation and function [7]. Innate immunity that includes NK cells, NKT cells, dendritic cells, and neutrophils are all critical in controlling the early primary infection [8]. Inflammatory cytokines produced by innate immune cells including TNF-α, IFN-γ, IL-12 and IL-18 are also important for curtailment of infection during the first week before onset of adaptive immunity [8,9].

The mechanisms of host response to infections under conditions of Selenium deficiency need to be understood to enable better management of epidemic outbreaks and to reduce the risk of this disease. In particular, very little is known about the specific host-pathogen interactions of intracellular bacteria with a Se-deficient host. In this study, Se-deficient C57BL/6 mice become susceptible to infection of *L. monocytogenes *due to impaired systemic innate immunity that is plausibly triggered by altered immune responsiveness to *L. monocytogenes *under the Se-deficient environment.

## Results

### Se-Deficient Mice Had Lower Amounts of Selenium and GPx

Se deficiency was performed by feeding 14 days pregnant female mice with Se deficient chow. Measurements of Se levels and of GPx activity in the group Se deficient Mothers confirmed the deficiency (data not shown), while Control Mothers and Control Offspring had mean levels of 231 ng and 228 ng Se/ml, 3.846 U and 3.872 U GPx/ml, in Se deficient Mothers and Offspring the values were 45 ng and 42 ng Se/ml, 1.128 U and 1.110 U GPx/ml (see Additional file 1), respectively. Se-deficient diets administered during mating, pregnancy and lactation led to a reduction of 5.4-and 3.4-times, respectively of Se and GPx in the mothers. The blood of all mice was collected and then the plasma was separated to measure the concentration of Se, MDA, activity of SOD, GSH-Px and CAT. There were extremely significant difference between the two groups (*p < 0.01*) in Se level and the activity of SOD, GSH-Px and CAT; but significant difference (*p < 0.05*) between the two groups in the concentration of MDA (see Additional file 1).

### Se-deficient mice exhibit increased bacterial burden in organs

**Figure 1:**
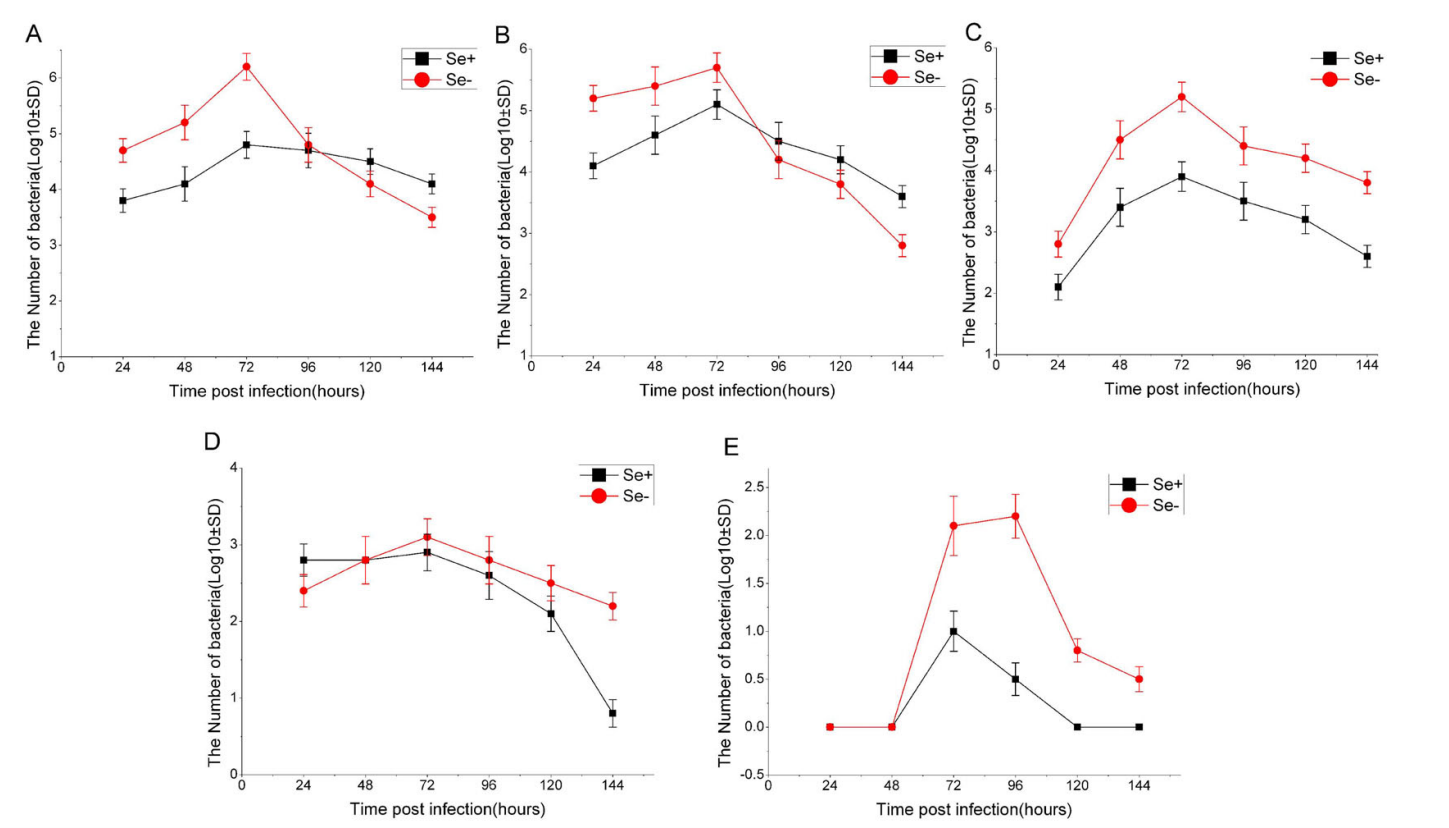
**Survival and growth of *L. monocytogenes *in organs of Se-adequate or Se-deficient C57BL/6 mice**. Survival and growth of *L. monocytogenes *in the intestine (A), lymph nodes (B), liver (C), spleen (D) and brain (E) of Se-adequate (Se+) and Se-deficient (Se-) inoculated orally with 5 × 10^9 ^CFU bacteria. Bacterial growth was followed in the organs at 24, 48, 72, 96,120 and 144 h post infection. Results are expressed as the mean concentration per group ± standard deviation (SD). *p < 0.05, **p < 0.01.

We performed oral inoculation of Se-adequate and Se-deficient C57BL/6 mice with *L. monocytogenes*. After infection via the oral route, and followed bacterial invasion in the small intestine (Figure 1A), translocation to mesenteric lymph nodes (Figure 1B) and spread to the liver (Figure 1C), spleen (Figure 1D) and brain (Figure 1E) of C57BL/6 mice over a 144 h period. We observed, at 24 h post infection of 5 × 10^9 ^CFU bacteria, a difference in the bacterial persistence of the Se-deficient mice in the intestine and mesenteric lymph nodes compared to the Se-adequate mice (Figure 1B). At 24 to 72 h post infection, multiplication of bacteria in the intestine, lymph node and the liver of Se-adequate mice were impaired (Figure 1A, B and 1C). Bacterial counts of the Se-adequate and Se-deficient mice were similar in the spleen at 48 h post infection (Figure 1D). Liver and brain of the Se-deficient mice were infected consistently at 72 to 120 h post infection compared to Se-adequate (Figure 1C and 1E).

### Se-deficient mice exhibit a decreased number of innate immune cell types in spleen

**Figure 2:**
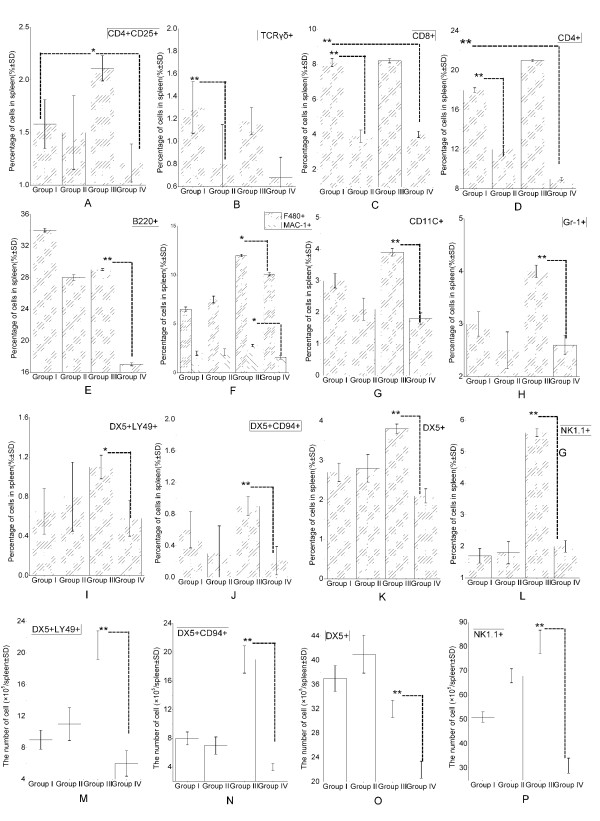
**Analysis of splenic cell populations in *L. monocytogenes *infection**. The populations of the NK cell subsets are indicated for the four groups; Group I, non-infected Se-adequate; Group II, non-infected Se-deficient; Group III, infected Se-adequate; and Group IV, infected Se-deficient. Cell surface expression of the indicated markers was determined for splenic lymphocytes from above four groups of mice on hour 72 of *L. monocytogenes *infection. Data are shown as percentage of positive cells in the spleen for each marker. Data are plotted as mean ± SD of analysis (50,000 events per sample) done on individual mouse spleen (*N *= 5 for each group). Statistical comparisons done between Se-adequate and Se-deficient groups indicated significant reduction in the expression of CD4 and CD8 under the conditions of Se deficiency. Statistical analysis done between Se-adequate and infected Se-adequate mice groups indicated significant increase in the expression of NK1.1 and DX5 post infection. Statistical analysis done between infected Se-adequate and infected Se-deficient mice groups indicated significant reductions in CD4+CD25+(A), TCRγδ+(B), CD8+(C), CD4+(D), B220+(E), F480+ and MAC-1+(F), CD11c+(G), Gr-1+(H), DX5+Ly49D+(I), DX5+CD94+(J), DX5+(K), and NK1.1+(L) populations under the conditions of Se deficiency. The percentages of DX5+Ly49D+(M), DX5+CD94+(N), DX5+(O), and NK1.1+(P) cells obtained after analysis of individual spleen samples were converted into numbers of each cell population based on the total splenocyte count obtained for each mouse. Results are expressed as the mean cell count per group ± standard deviation (SD). *p < 0.05, **p < 0.01.

To address the mechanism(s) responsible for the decrease of host resistance to *L. monocytogenes *infection under the conditions of Se deficiency, we evaluated the phenotype and numbers of the various immune cell types in the spleen (Figure 2). Four groups of mice were included in the study: Group I, non-infected Se-adequate; Group II, non-infected Se-deficient; Group III, infected Se-adequate; and Group IV, infected Se-deficient. Most striking was the significant increase in NK1.1+ and DX5+ cells (*P < 0.01*) in infected Se-adequate mice (Group III), indicative of a rapid onset of NK cell response to infection which was lacking in infected Se-deficient mice (Group IV) (Figure 2K and 2L). Both subsets of DX5+CD94+ (P < 0.01) and DX5+ Ly49D+ (P < 0.05) were lower in infected Se-deficient mice (Group IV) (Figure 2I and 2J). *L. monocytogenes *infection of Se-deficient mice (Group IV) induced decreased percentages of many innate immune cell types defined by the expression of F480 (*p < 0.01*), MAC-1(*p < 0.05*), CD11c (*p < 0.01*), and Gr-1 (*p < 0.01*) (Figure 2F,G and 2H). In comparison to non-infected Se-adequate (Group I) mice, Group II mice in the absence of any infection exhibited significantly reduced percentages of CD4+ and CD8+ T cells (*p < 0.01*) (Figure 2A,B,C,D). The overall numbers (Figure 2P) of NK cells and their subsets in the spleen were also markedly decreased in infected Se-deficient (Group IV) mice, reiterating the lack of effective NK response against *L. monocytogenes *infection under the conditions of Se deficiency. The number of DX5+LY49+, DX5+CD94+ and DX5+ cells in the spleen was significantly decreased in infected Se-deficient (Group IV) mice in comparison to infected Se-adequate (Group III) (*p < 0.01*) (Figure 2M,N and 2O). Infected Se-deficient (Group IV) mice also showed significant reductions in the percentages of dendritic cells, neutrophils, and TCR-γδ+ T cells as evident from the decreased splenic expression of CD11c, Gr-1, and TCR-γδ, respectively (Figure 2B,G and 2H). In total, the overall numbers of these innate immune cell subsets also supported their decrease in the spleen. Infected Se-deficient (Group IV) mice exhibited lower percentages of MAC-1+ cells (Figure 2F). Thus, whether macrophage numbers are modulated in infected Se-deficient mice (Group IV) is unclear. The percentages of T and B cells defined by the expression of CD4 (*p < 0.01*), CD8 (*p < 0.01*), CD25 (*p < 0.05*), and B220 were also lower in infected Se-deficient (Group IV) mice in comparison to non-infected Se-adequate (Group I) (Figure 2A,C,D and 2E). However, as T cells showed a constitutive reduction in Se-deficient mice even in the absence of infection, it was deduced that *L. monocytogenes *did not cause any further reduction of adaptive immune cell types. Furthermore, effects of *L. monocytogenes *infection were studied at 72 hour post infection, an early time point when adaptive immunity may be expected to play little role. In summary, it appeared that the ability of the host to elicit a rapid recruitment and activation of systemic innate immune response to infection was severely compromised under the conditions of Se deficiency.

### Se-deficient mice exhibit reduced splenic NK cytotoxicity

**Figure 3:**
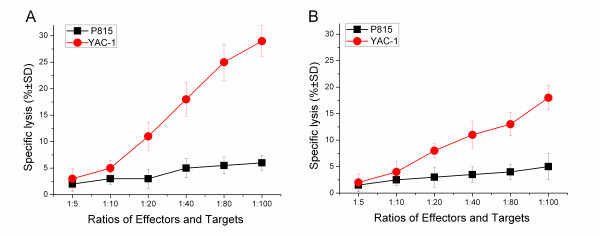
**Splenic NK cytotoxicity after *L. monocytogenes *infection**. C57BL/6 mice were infected with *L. monocytogenes *in the Se-adequate or Se-deficient state. At 72 h post infection, splenocytes were obtained from each group mice, and their ability to kill NK-sensitive YAC-1 target cells, and NK-insensitive P815 target cells was determined by ^51^Cr-release assay. Percentage-specific killing ± SD at the various ratios of effectors and targets are indicated for spleen cells from infected Se-adequate (A) and infected Se-deficient mice (B).

NK cells are the first line of defense against many infections, and are capable of mounting a cytotoxic response against infected target cells. Se-adequate and Se-deficient mice were challenged with *L. monocytogenes*, and the ability of splenic effectors to kill NK sensitive targets was evaluated at 72 hour post infection. Splenic effectors from infected Se-adequate mice killed YAC-1 targets effectively, and exhibited negligible killing on NK-insensitive P815 target cells (Figure 3A). In contrast, splenic effectors from infected Se-deficient mice exhibited weak killing of YAC-1 target cells (Figure 3B). In total, the level of NK cytolytic activity on 72 hour of *L. monocytogenes *infection in infected Se-deficient mice was significantly lower than that of infected Se-adequate mice. So the difference in levels of killing could be due to reduced NK numbers in the spleens or a lower cytotoxic ability of the NK cells.

### Levels of cytokine in the serum samples of Se-deficient mice

**Figure 4:**
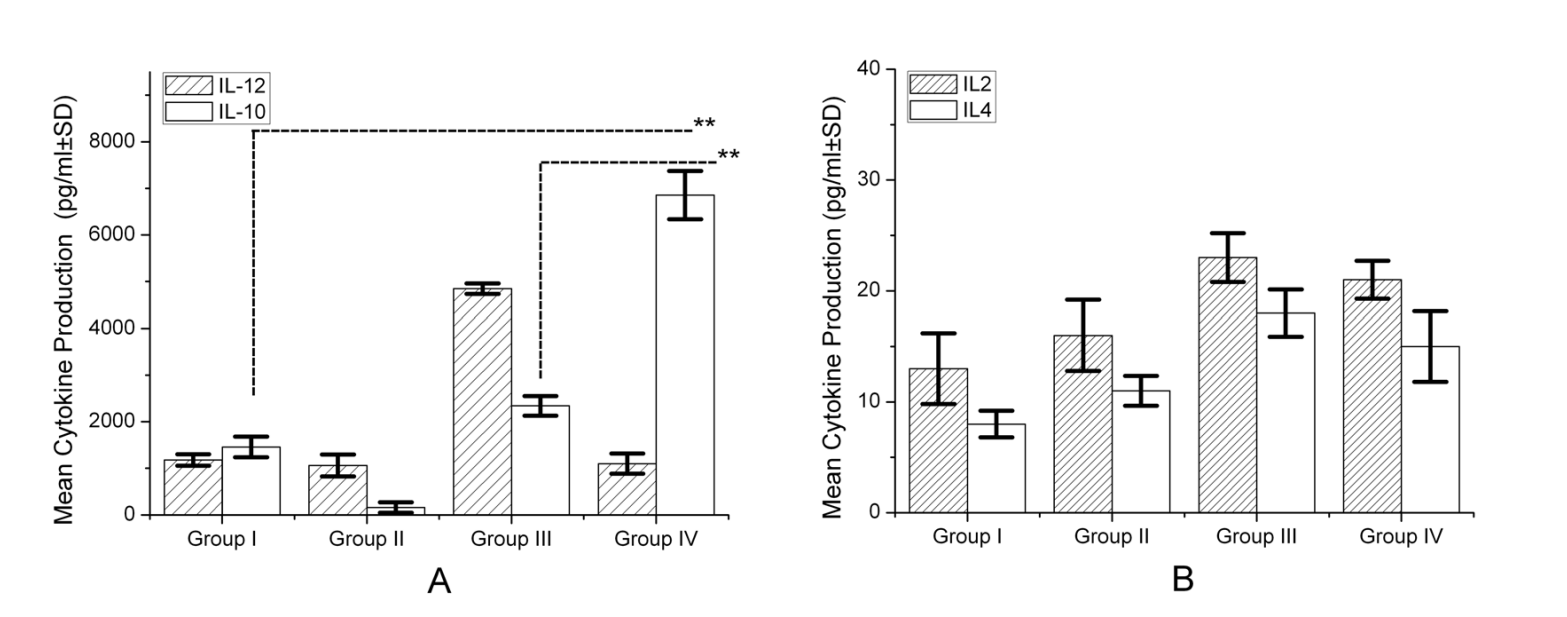
**Serum cytokine levels in response to *L. monocytogenes *infection**. Individual serum samples were assayed for levels of IL-12 and IL-10 (A), IL-2 and IL-4 (B). The levels of IL-12 were significantly lower in infected Se-deficient mice in comparison to infected-adequate mice. Results are expressed as the mean concentration per group ± standard deviation (SD). *p < 0.05, **p < 0.01.

In comparison to non-infected Se-adequate mice, *L. monocytogenes *infection evoked increased production of serum IL-12 at 72 h post infection in infected Se-adequate mice. However, this increase in serum IL-12 did not occur in infected Se-deficient mice (group IV) (Figure 4A). In contrast, serum IL-10 levels were elevated in infected Se-deficient mice (group IV) in comparison to infected Se-adequate (group III) or Se-adequate controls (group I) (*p < 0.01*) (Figure 4A). Thus, *L. monocytogenes *infected Se-deficient mice exhibited a skewed peripheral inflammatory response characterized by overt production of IL-10 and decreased production of IL-12. IL-2 and IL-4 levels demonstrated insignificant differences between Se-adequate and Se-deficient mice (Figure 4B). In contrast, serum levels of IL-2, IL-4, IFN-γ, IL-6 and IL-1β were similar in both infected Se-adequate and Se-deficient mice at 72 h post infection, however TNF was not detected in the serum of any of the groups (data not shown).

### Cytokine expression in the livers of Se-deficient mice could be modulated by *L. monocytogenes *infection

**Figure 5:**
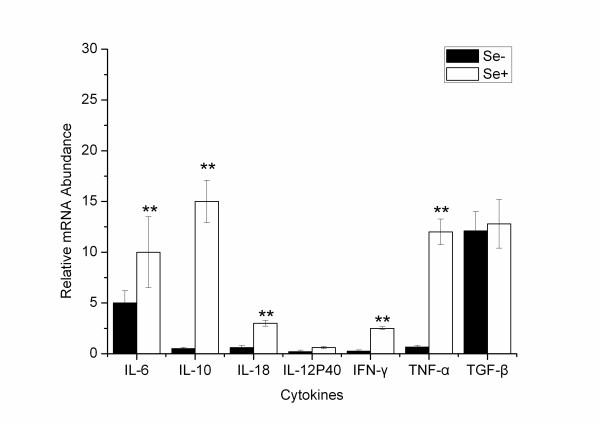
**Liver cytokine expression in infected Se-deficient or adequate mice**. Cytokine mRNA expression was determined in liver samples from each Se-deficient mice. C57BL/6 mice were infected with *L. monocytogenes*. Livers were obtained from infected Se-adequate and Se-deficient mice at 72 h post infection. Data are presented as relative mRNA abundance normalized to levels of *β-actin*. Mean ± SD of expression levels for *N *= 5 in each infected group is indicated. *p < 0.05, **p < 0.01.

As *L. monocytogenes *infection was causing a dramatic increase in the number of bacteria in the organs of Se-deficient mice, we sought to ascertain the relative levels of inflammatory (IL-6, IL-18, TNF-α, IL-12p40, and IFN-γ) and anti-inflammatory cytokines (IL-10 and TGF-β) in the livers. This was achieved by performing quantitative RT-PCR on samples from Se-deficient and Se-adequate mice and normalizing values to expression levels of *β-actin*. Livers from *L. monocytogenes *infected Se-adequate mice showed a significant increase in the expression of cytokines IL-6, TNF-α, IL-18, and IL-10 relative to expression levels in the liver of Se-deficient mice (*p < 0.01*) (Figure 5). IFN-γ expression levels also showed an increased trend in the livers of Se-adequate mice (*p < 0.01*). In contrast, the expression levels of IL-12p40 and TGF-β were similar in liver of the two groups.

## Discussion and Conclusion

Dietary selenium (Se) is an essential micronutrient that affects various aspects of human health, including optimal immune responses. However, in some regions with lower soil selenium contents, the increasing number of some diseases in humans was observed in association with selenium deficiency [10,11]. Se deficiency is thought to contribute to disease by making the body more susceptible to nutritional and biochemical stresses as well as infectious diseases. Selenium deficiency cause directly or indirectly Keshan Disease, Kashin-Beck Disease, Myxedematous Endemic Cretinism, and Influenza [3]. In this study, we measured selenium levels, cytokine expression levels, antioxidant enzyme activity, and bacterial burden in organs after Se deficient C57BL/6 mice were infected with *L. monocytogenes*. We found the decrease of C57BL/6 mice resistance to infection is associated with the reduced antioxidant enzyme activity and decreased innate immune responses.

The fundamental importance of Se to optimal immune function has been widely demonstrated. An adequate Se intake is essential for an appropriate immune response to various infectious diseases. Se supplementation results in more rapid poliovirus clearance [12] as well as lowered hospitalization rates among human immunodeficiency virus (HIV)-infected patients [13]. Se deficiency is associated with Keshan disease, a disease correlated with coxsackievirus infection of the heart muscle [14]. In our present study, we observed a difference in the bacterial persistence of the Se-deficient mice in the intestine and mesenteric lymph nodes compared to the Se-adequate mice at 24 to 72 h post infection of 5 × 10^9 ^CFU bacteria. In addition, liver and brain of the Se-deficient mice were infected consistently at 72 to 120 h post infection compared to Se-adequate. These results suggest that Se deficiency is implicated probably in playing roles both in the crossing of the intestinal barrier and in the early or later stages of the *L. monocytogenes *infection process.

Comparative analysis was conducted between the Se-deficient and Se-adequate mice following the infection of *L. monocytogenes *(see additional file 1). Overall results showed that the activity of antioxidant (MDA, SOD, GSH and CAT) in Se-adequate mice was significantly higher than that in Se-deficient mice following the infection of *L. monocytogenes*. It is suggested that the relativity might exist between Se deficiency and infection of *L. monocytogenes*. Ribeiro et al. [15] suggested a decrease in GSH levels in host might render them susceptible to *schistosomula*. It may explain the susceptibility of host to infection of *L. monocytogenes *due to partly a decrease in GSH levels of Se deficient mice in this study. In general, the roles of selenoenzymes, GPx, in the intracellular and extracellular antioxidant defense systems have been well characterized. GPx isoenzymes use GSH as a donor of reducing equivalents to detoxify H_2_O_2_, peroxi-derivatives of various organic compounds (e.g., fatty acids, cholesterol, and phospholipids) [16], and peroxynitrite [17]. Thus, GPx is essential for maintenance of the cellular redox status and for the regulation of the redox-sensitive signaling and transcription mechanisms. Notably, although Se deficiency has been shown to suppress GPx expression and decrease the corresponding enzymatic activities, a similar phenomenon was also observed in this study. Selenium deficiency could lead to enhanced lipid peroxidation through loss of selenium-dependent glutathione peroxidase activity [18]. Therefore, effects of Se deficiency on immune function may not be solely explained by the change in GSH levels.

In order to evaluate the effect of Se on immune cell types, another experiment was conducted to analyze the phenotype and numbers of the various immune cell types in the spleen of *L. monocytogenes *infection under the conditions of Se deficiency. Most striking was the significant increase in NK1.1^+ ^and DX5^+ ^cells, indicative of a rapid onset of NK cell response to infection which was lacking in infected Se-deficient mice. Both subsets of DX5^+^CD94^+^and DX5^+^Ly49D^+ ^were also relatively lower in infected Se-deficient mice. *L. monocytogenes *infection of Se-adequate mice induced increased percentages of many innate immune cell types defined by the expression of F480, MAC-1, CD11c, and Gr-1 (macrophages, dendritic cells, and neutrophils) compared to infected Se-deficient mice. In comparison to Se-adequate mice, Se-deficient mice in the absence of any infection exhibited significantly reduced percentages of CD4+ and CD8+ T cells, whereas differences in other cell types were insignificant. The overall numbers of NK cells and their subsets in the spleen was also markedly decreased in infected Se-deficient mice, reiterating the lack of effective NK response against *L. monocytogenes *infection under the conditions of Se deficiency. In total, the overall numbers of these innate immune cell subsets also supported their decrease in the spleen. The percentages of T and B cells defined by the expression of CD4, CD8, CD25, and B220 were also lower in infected Se-deficient mice in comparison to non-infected Se-adequate mice. However, as T cells showed a constitutive reduction in Se-deficient mice even in the absence of infection, it was deduced that *L. monocytogenes *did not cause any further reduction of adaptive immune cell types. Furthermore, effects of *L. monocytogenes *infection were studied at 72 hour post infection, an early time point when adaptive immunity may be expected to play little role. In summary, it appeared that the ability of the host to elicit a rapid recruitment and activation of systemic innate immune response to infection was to certain extent compromised under the conditions of Se deficiency. It has been proved that Se is involved in regulating oxidative stress, redox, and other crucial cellular processes in nearly all tissues and cell types, including those involved in innate and adaptive immune responses [19-21]. A number of studies in agricultural animals have provided insight into the effects of Se deficiency or Se-supplementation on immune responses [21]. In most cases, these studies have demonstrated an enhancement of both cell-mediated and humoral immune responses by increasing levels of Se intake. In experimental animal studies, Se-deficiency has been shown to result in less robust immune responses to viruses, tumors, and allergens, compared to Se-adequate controls [3]. However, the results are less clear regarding the benefits of Se-supplementation above adequate levels in conferring additional immunological protection. Limited data from studies in humans suggest that Se-supplementation may enhance immunity, including both humoral and cell-mediated responses [22]. However, it remains unclear whether all types of immune responses are reduced by decreasing Se intake.

Se may influence cytokine production of innate immune. We measured cytokine levels in the serum of each group mice. IL-12 production is important for influencing the function of many cell types including dendritic cells, NK cells, and T cells. In comparison to non-infected Se-adequate mice, *L. monocytogenes *infection evoked increased production of serum IL-12 at 72 h post infection in infected Se-adequate mice. However, this increase in serum IL-12 did not occur in infected Se-deficient mice. In contrast, serum IL-6 levels were elevated in infected Se-deficient mice in comparison to infected Se-adequate or Se-adequate controls. *L. monocytogenes *infected Se-deficient mice exhibited a skewed peripheral inflammatory response characterized by overt production of IL-10 and decreased production of IL-12. IL-2 and IL-4 levels demonstrated insignificant differences between Se-adequate and Se-deficient mice. Levels of IL-2 and IL-4 in Se-deficient mice were similar compared to Se-adequate mice at 72 h post infection. Serum levels of IFN-γ, IL-6 and IL-1β were shown also similar in both infected Se-adequate and Se-deficient mice at 72 h post infection, however TNF was not detected in the serum of any of the groups. A study in mice involving Se-deficiency and the gastrointestinal parasite, *Heligmosomoides polygyrus*, showed that low dietary Se resulted in decreased resistance to the nematodes upon secondary infection [23]. The lowered resistance did not appear to involve IL-4, as Se-deficiency had no effect on circulating levels of this key cytokine involved in host protection. Maintenance of non-infected Se-deficient mice is associated with liver secretion of anti-inflammatory cytokines IL-10 [24] and TGF-β. As *L. monocytogenes *infection was causing a dramatic increase in the number of bacteria in the organs of Se-deficient mice, we measured the relative levels of inflammatory (IL-6, IL-18, TNF-α, IL-12p40 and IFN-γ) and anti-inflammatory cytokines (IL-10 and TGF-β) in the livers by using quantitative RT-PCR on samples from each Se-deficient mice and normalizing values to expression levels of *β-actin*. Livers from infected Se-deficient mice showed a significant increase in the expression of cytokines IL-6, TNF-α, IL-18, and IL-10 relative to expression levels in the liver of non-infected Se-deficient mice. IFN-γ expression levels also showed an increased trend in livers of infected Se-deficient mice. In contrast, the expression levels of IL-12p40 and TGF-β were similar in liver of the two groups. Liver cytokine expression also suggested overt production of IL-10 in Se-adequate hosts. Infection with *L. monocytogenes *induces a strong T-helper type 1 (Th1) response, resulting in expansion of *L. monocytogenes *specific CD8+ T cells, which play a significant role in bacterial clearance. However, the Se-deficient mice had a more T helper type 2 (Th2)-like pattern of cytokine expression. IL-2 stimulates T-cell activation and expansion [25,26], especially the development of Th2 cells by stabilizing the accessibility of the IL-4 gene [27]. In vivo, IL-2 neutralization inhibits IL-4 production [27].

GSH levels in the immune cells may play a pivotal role in determining the Th1/Th2 balance. GSH depletion in antigen presenting cells (APCs) shifts the immune response toward a Th2 response both in vitro and in vivo [28]. The intracellular GSH levels have also been correlated with the Th1 cytokine production versus the Th2 cytokine production by macrophages and CD4+T cells [29]. The effect of GSH levels on Th1/Th2 balance is partly mediated by IL-12 and IL-4 [28,29]. Our data support these studies by showing a higher level of IL-12 expression in Se-adequate mice. Although a difference in IL-4 expression between Se-adequate and Se-deficient mice was not observed in our study, the diminished Th1 response in Se-deficient mice may be explained by the changes in production of other chemokines and cytokines by APCs and macrophages, such as IL-18.

While higher levels of Se have generally been shown to provide protection against viruses, the relationship between host Se status and immunity to bacterial pathogens is not so one-sided [3]. Most of the available data suggest the influence of Se status on resistance to infections with bacteria, parasites and fungi, indicates different results. For example, an early study involving *Candida albicans *infection in mice showed that Se-deficiency resulted in higher susceptibility to this pathogen [30]. The authors demonstrated an impaired ability of Se-deficient, glycogenelicited peritoneal neutrophils to kill *C. albicans *in vitro, which was repeated in other experiments and shown to be due to reduced superoxide production, oxygen consumption and glucose utilization [31]. In contrast, killing of *Salmonella typhimurium *and *Staphylococcus aureus *by neutrophils was unaffected by Se-deficiency [30]. Se-deficient rats exhibited increased mortality after i.p. injection of *S. aureus*, although injection of lower doses of organisms did not result in mortality differences when comparing Se-deficient and -adequate rats. In contrast, Se-deficiency was shown to increase survival of rats infected with pathogens such as *S. typhimurium*, *Plasmodium bergeii*, or *L. monocytogenes *[32,33]. Se-deficient mice infected with *Trypanosoma musculi*, a protozoan parasite specific to mouse, cleared the pathogen whereas mice fed Se-adequate or -supplemented chow exhibited sustained parasitemia [34]. A possible explanation was offered by the observation that trypanosomes lack sufficient capacity to reduce oxidative stress because the parasites do not have the classical glutathione peroxidase (GPX)/GR enzymatic system [35], thus making them reliant on Se-sufficient host antioxidants for survival. Moreover, studies in mice infected with *T. cruzi *demonstrated that Se-deficiency in the murine host increased the severity of *T. cruzi*-induced chronic inflammatory myopathy [36]. In our study, despite the increasing burden in intestine, liver and brain in infected Se-deficient mice, mice were not observed dead throughout the experiments. Overall, it is explained that the increased the number of bacteria in tissues found in this study were due to inadequate immune responses or increased oxidative stress and inflammation in these tissues.

In summary, Se-deficient mice had an altered innate immune response to *L. monocytogenes *infection. Our results suggest that the outcome of *L. monocytogenes *infection of Se-deficient mice is associated with Se status of host. Further studies will focus on mechanistic studies that involve specific selenoproteins and the roles they play during inflammation and immune responses not only because many selenoproteins play critical roles in reducing oxidative stress and balancing redox in a wide variety of tissues and cell types, including those involved in innate and adaptive immune responses but also because the functions for many selenoproteins remain unknown.

## Methods

### Bacteria

*L. monocytogenes *strain 4b used in this study were purchased from China Institute of Veterinary Drug Control. Briefly, bacteria were grown in liquid culture in brain-heart infusion (BHI) medium (Gibco). At mid-log phase (OD_600 _> 0.8) bacteria were harvested and frozen at -80°C (in 20% glycerol). CFU were determined by performing serial dilutions in 0.9% NaCl, which were spread on BHI agar plates.

### Selenium depletion and measurement of antioxidant enzymeactivities

Female 8- to 10-wk-C57BL/6 mice were obtained from the Centre of Beijing Experimental animals. All animal studies complied with the Guidelines for Ethical Conduct in the Care and Use of Experimental Animals and approved by Ethics Committees on Animal Experimentation in Institute of Zoology, People's Republic of China. Mice were maintained in the animal facility at the Institute for Zoology (Chinese Academy of Sciences, Beijing, PR China). Se depletion was conducted according to Moreno-Reyes and Andrea P. de Souza protocol [37,38]. Briefly, mice were depleted in Se since the embryonic development, by feeding female C57BL/6 mice during pregnancy and lactation periods with diets containing different levels of Se. Both diets have all the recommended nutrients, such as vitamins, amino acids (52.5 g/kg), oligoelements and fatty acids (41.2 g/kg), differing only in the Se levels: 0.2 mg Se/kg (control diet with adequate Se level), and 0.005 mg Se/kg (Se-deficient). After weaning (21 days after birth), groups Control Mothers (n = 30) and groups Se deficient Mother (n = 30) were sacrificed and the plasma and organs collected. The female offspring (30 Se-adequate mice and 30 Se-deficient mice) were separated throughout the experiment.

Approximately 30 μl blood samples were collected in heparinized microcapillaries from the tail of each mouse prior to infection. Plasma samples were obtained after centrifugation of blood in a micro-hematocrit centrifuge and were stored at -20°C until analysis. The plasma levels of Se were measured by in Zeeman corrected atomic absorption spectrometry with a limit of sensitivity of 64 nM (5 ng/mL) according to method [39]. Undetectable concentrations were assigned a value of 5 ng/mL [37].

The plasma samples for analysis of antioxidant enzyme activities were performed according to protocols reported previously. Briefly, GPx activity was measured by a coupled assay with yeast GR, using H_2_O_2 _as a substrate [39]. CAT activity was determined by the method of Aebi that monitors the rate of H_2_O_2 _decomposition [40]. The total SOD activity was determined by the nitroblue tetrazolium assay of Spitz and Oberley [41]. Malondialdehyde (MDA) concentration was measured by spectrophotometry with thiobarbituric acid [42].

### Inoculation of *L. monocytogenes*

For infection, frozen stocks of bacteria were thawed and diluted in 0.9% sterilized physiological saline. For mice, 5 × 10^9 ^CFU mixed with PBS 150 mg/ml CaCO_3 _were injected intragastrically to Se-adequate or Se-deficient mice starved for 24 h. Viable bacterial counts within intestine, mesenteric lymph nodes, spleen, liver, and brain were determined by homogenizing the tissue in PBS containing 0.05% Triton X-100 and plating on brain-heart infusion (BHI) agar plates (Gibco). The number of intestinal *L. monocytogenes *organisms was determined by sequential dissociation of intestinal tissues and plating on BHI plates containing streptomycin (100 mg/ml) and nalidixic acid (50 mg/ml) to inhibit the growth of endogenous bacterial flora. *L. monocytogenes *colonies were identified by their characteristic morphology and by Gram staining.

### Bacterial burden in organs

At 24, 48, 72, 96, 120 and 144 h after infection, mice were euthanized by CO_2 _asphyxiation and the organs (the whole mesenteric lymph nodes, liver, spleen and brain) were aseptically dissected. The small intestine was rinsed and incubated for 2 h in 100 mg/l gentamicin to kill extracellular bacteria from the intestinal lumen. For the spleen, single-cell suspensions were obtained by squishing the organ between frosted ends of a glass slide. Liver and brain were homogenized using a motorized homogenizer. An aliquot of the cell suspension or homogenate was lysed with water for 30 s, and then evaluated for the numbers of viable bacteria. Ten-fold serial dilutions of the tissue homogenates, in 100 μl volume of 0.9% NaCl were plated on Brain Heart Infusion broth agar (Gibco). For assessing bacterial burdens at each time points after infection, the entire volume of tissue homogenate was spread onto several plates. Colonies were counted after 24 h of incubation at 37°C.

### Flow cytometric analysis of immune cell subsets

Single-cell suspensions of spleens were analyzed for the various immune cell types based on their surface expression of various markers. For staining with all Abs, cells were first incubated on ice (10^6 ^cells in 100 μl of PBS plus 1% BSA) with anti-mouse CD32/CD16 (Fcγ II/III receptor). After 10 min, 3-5 μl of different FITC- or PE-labeled anti-mouse Abs were added and incubated for an additional 30 min on ice. Abs against the following cell surface markers were used to identify the various immune. Cell types: B220, CD4, CD8, TCR-γδ, CD25, F480, MAC-1, CD11c, Gr-1, NK1.1, DX5, CD94, and Ly49D. All Abs were purchased from Biolegend. After 30 min, cells were washed and fixed in 1% formaldehyde in PBS and acquired on an ACCURI C6 Flow Cytometer (America). Analysis was done using ACCURI software (America).

### NK cytotoxicity

Cytotoxicity of NK cells in vitro was assessed in a ^51^Cr-release assay on YAC-1 target cells. Murine NK-sensitive target cells (YAC-1 cell) as well as NK-insensitive cells (P815 cell) were propagated in RPMI 1640 medium additionally supplemented with 10% FBS and 10 μg/ml gentamicin 37°C, 5%CO_2_. Both cell lines were purchased from the China Type Culture Collection. For the assay, 5 × 10^6 ^target cells were labeled with 50 μCi ^51^Cr (Amersham Pharmacia Biotech) for 1 h at 37°C and washed three times. Effector cells were prepared by tweezing the spleens between the frosted ends of two sterile glass slides in R8 medium. Cells were subsequently passed through Falcon 2360 cell strainers (BD), centrifuged, and resuspended in R8 medium. Various ratios of effectors and targets were co-cultured for 4 h at 37°C in 96-well round bottom culture plates (Costar). The supernatants were collected, and radioactivity was detected by gamma counting. The percentage of specific lysis was calculated using the formula: 100 × ((experimental cpm-spontaneous cpm)/(total cpm-spontaneous cpm)). The percentage-specific killing obtained at various E:T ratios was also converted to lytic units, wherein 1lytic unit represents the number of effectors yielding 15% specific lysis of 2.5 × 10^4 ^YAC-1 targets.

### Cytokine ELISAs

Blood samples were obtained by cardiac puncture under euthanized by CO_2 _asphyxiation and collected in microtainer serum separator tubes. The serum was separated by quick high-speed centrifugation at 4°C, and then stored at -80°C. Levels of serum IL-12, IL-6, IL-2, IL-4, IFN-γ, IL-1β and IL-10 were assayed by sandwich ELISA kit purchased from Biolegend. Cytokine standards were also purchased from Biolegend. Duplicate standard curves encompassing several doubling dilutions of the standard were included on each plate. All serum samples were assayed at the same time in cytokine detection.

### Liver cytokine expression by quantitative RT-PCR

Livers from individual mice were dissected out, pooled, and snap-frozen in a dry ice/100% ethanol bath. Total RNA was extracted using the Qiagen RNeasy Mini kit according to the instructions of the manufacturer along with rapid mechanical lysis. Briefly, livers were homogenized using a motorized homogenizer and lysed in 1 ml of lysis buffer in a MiniBeadbeater 3110BX (BioSpec Products) with glass beads. Total RNA from homogenates was extracted and treated with RNase-free DNase I (Roche Applied Science) for 30 min at 37°C. DNase was then removed according to the instructions of the manufacturer. A total of 2-5 μg of total RNA was taken for cDNA synthesis.

cDNA was synthesized using AncT primers (Invitrogen). RNA was made linear at 65°C for 5 min and cDNA was synthesized in a 40 μl reaction volume containing: 1.5 μl of AncT primers (100 pM/μl), 8 μl 5×first-strand buffer, 4 μl of DTT (100 mM), 5 μl of dNTP (5 mM), 1 μl of RNase OUT (40 U/μl), 2 μl of Superscript II (200 U/μl) (Invitrogen), and 15 μl of RNA template. Reverse transcription was performed in a Thermo Cycler 9700 (Applied Biosystems) at 42°C for 15 min and 45°C for 2 h. Identical samples not treated with Superscript II were also prepared as controls to measure DNA contamination. The remaining RNA template was hydrolyzed with 1 M NaOH at 65°C for 5 min and neutralized with 1 M HCl. cDNA was purified using Microcon YM-30 centrifugal filter units (Millipore). The number of amplicons was measured by quantitative real-time PCR using gene-specific primers and quantitative PCR SYBR green supermix (ABgene). Primers were designed using Primer Express 2.0. The primer pairs used are listed in seeing additional file 2. *β-actin *was used as an internal reference control. Ten-fold dilutions of cDNA were used as template to generate the standard curve for each primer-template set (1×, 1/10×, 1/100× and 1/1000×). This standard curve was run together with triplicate reactions of the uncharacterized samples. PCR was performed in sealed tubes in a 96-well microtiter plate in an ABI Prism 7000 Thermocycler (Applied Biosystems). The 25 μl reaction consisted of 12.5 μl of quantitative PCR SYBR green supermix, 2.5 μl of primer mix (1.5 pM/μl each), and 10 μl of template. Thermal conditions were as follows: activation at 95°C for 15 min, followed by 40 cycles of denaturation at 95°C for 15 s, annealing at 60°C for 1 min, and extension at 72°C for 1 min. Fluorescence was measured during the annealing step and plotted against the amplification cycle. Relative quantitative analysis of the data was extrapolated from the standard curve.

### Statistical analysis

All values are expressed as mean ± standard deviations (SD). All the data are analyzed via ANOVA, as appropriate, and stated in the figure legends were used to determine the statistical significance of the experimental data.

## Abbreviations

(MDA): Malondidehyde; (SOD): Superoxide dismutase; (GPx): glutathione peroxidase; (CAT): Catalase.

## Authors' contributions

WCM carried out determination of Selenium, GPx, cytokine detection, cytokine expression level in lever, and drafted the manuscript. WHJ carried out subsets of spleen cell. LJ participated in inoculation of bacteria. YH participated in determination of bacterial burden in organs. WL performed the statistical analysis. HHX conceived of the study. DMX participated in its design and coordination and helped to draft the manuscript. All authors read and approved the final manuscript.

## Supplementary Material

Additional file 1 **Se level and antioxidant enzyme activity in plasma of Se-deficient and-adequate mice**. The blood of all mice (Se-deficient mice = 30; Se-adequate mice = 30) was collected and then the plasma were separated to measure concentration of Se, MDA, activity of SOD, GSH-Px and CAT. There were extremely significant difference between the two groups (*p < 0.01*) in Se level and the activity of SOD, GSH-Px and CAT; but significant difference (*p < 0.05*) between the two groups in the concentration of MDA Results are expressed as the mean concentration per group ± standard deviation (SD). *p < 0.05, **p < 0.01. Click here for file

Additional file 2 **Table s1**. Primer sequences used for assay of liver cytokines. Click here for file
