# Supplementary material for: Selenium deficiency impairs host innate immune response and induces susceptibility to Listeria monocytogenes infection
Source: BMC Immunol. 2009 Oct 24;10:55. doi: 10.1186/1471-2172-10-55 (PMC2774297; doi:10.1186/1471-2172-10-55)
Supplement: Additional file 1 — Se level and antioxidant enzyme activity in plasma of Se-deficient and-adequate mice. The blood of all mice (Se-deficient mice = 30; Se-adequate mice = 30) was collected and then the plasma were separated to measure concentration of Se, MDA, activity of SOD, GSH-Px and CAT. There were extremely significant difference between the two groups (p < 0.01) in Se level and the activity of SOD, GSH-Px and CAT; but significant difference (p < 0.05) between the two groups in the concentration of MDA Results are expressed as the mean concentration per group ± standard deviation (SD). *p < 0.05, **p < 0.01. [file 1471-2172-10-55-S1.DOC]

**Table S2. Se level and antioxidant enzymes activity in plasma of Se-deficient and-adequate mice.** The blood of all mice (Se-deficient mice=30; Se-adequate mice=30) were collected and then the plasma were separated to measure concentration of Se, MDA, activity of SOD, GSH-Px and CAT. There were extremely significant difference between the two groups (p<0.01) in Se level and the activity of SOD, GSH-Px and CAT; but significant difference (p<0.05) between the two groups in the concentration of MDA Results are expressed as the mean concentration per group ± standard deviation (SD). **p<0.05, **p<0.01*.

| **Mice group** | **Se （μmol/L）** | **SOD(U/ml)** | **GSH-Px (U/L)** | **CAT(U/ml)** | **MDA(nmol/ml)** |
| --- | --- | --- | --- | --- | --- |
| Se+ | 228±10.249** | 61.805±1.500** | 3940±147.126** | 7.379±0.125** | 4.852±0.143* |
| Se- | 42±3.983 | 37.638±1.577 | 1010±34.794 | 2.838±0.155 | 5.213±0.135 |
