# Supplementary material for: Selenium deficiency impairs host innate immune response and induces susceptibility to Listeria monocytogenes infection
Source: BMC Immunol. 2009 Oct 24;10:55. doi: 10.1186/1471-2172-10-55 (PMC2774297; doi:10.1186/1471-2172-10-55)
Supplement: Additional file 2 — Table s1. Primer sequences used for assay of liver cytokines. [file 1471-2172-10-55-S2.DOC]

Table.S1. Primer sequences used for assay of liver cytokines

| **Gene** | **Forward Primer(5’-3’)** | **Reverse Primer(5’-3’)** |
| --- | --- | --- |
| *TGF-*β | AAACGGAAGCGCATCGAA | GGGACTGGCGAGCCTTAGTT |
| *IL-10* | CAGCCGGGAAGACAATAACTG | CCGCAGCTCTAGGAGCATGT |
| *IL-6* | CCAGAAACCGCTATGAAGTTCCT | CACCAGCATCAGTCCCAAGA |
| *IL-12p40* | CCCCCAAAAGCTGTCTTCTG | GCAAAGGTGTCATGATGAACTTAGG |
| *IFN -*γ | ACAATGAACGCTACACACTGCAT | TGGCAGTAACAGCCAGAAACA |
| *IL-18* | AAGAAAGCCGCCTCAAACCT | TCTGACATGGCAGCCATTGT |
| *TNF-*α | ATCCGCGACGTGGAACTG | ACCGCCTGGAGTTCTGGAA |
| *β-actin* | TGACCGAGCGTGGCTACA | TCTCTTTGATGTCACGCACGAT |
